# Addition of bevacizumab to gefitinib plus chemotherapy as first-line therapy in EGFR L858R mutate advanced non-small cell lung cancer patients

**DOI:** 10.3389/fphar.2025.1503171

**Published:** 2025-10-08

**Authors:** Lingli Lei, Xiang Zhan, Jixian Li, Yi Ding, Jiaxin Li, Fengge Zhou, Alei Feng, Xiaomei Li, Zhe Yang

**Affiliations:** ^1^ Shandong Provincial Hospital, Shandong University, Jinan, Shandong, China; ^2^ Department of Radiotherapy, the Second Hospital of Hebei Medical University, Jinan, Shandong, China; ^3^ Tumor Research and Therapy Center,Shandong Provincial Hospital Affiliated to Shandong First Medical University, Jinan, Shandong, China

**Keywords:** non-small cell lung cancer, epidermal growth factor receptor, bevacizumab, gefitinib, prognosis

## Abstract

**Background:**

For advanced non-small cell lung cancer patients with the epidermal growth factor receptor L858R mutation, the efficacy of the combination of tyrosine kinase inhibitor (TKI) and chemotherapy is suboptimal. Currently, it is unclear whether the combination of bevacizumab, gefitinib, and chemotherapy could improve the survival.

**Materials and methods:**

Data was retrospectively collected at Shandong Provincial Hospital between June 2019 and December 2022. The patients were divided into two groups, ATC group receiving bevacizumab, gefitinib, and chemotherapy, and TC group receiving gefitinib and chemotherapy. After propensity score matching (PSM), progression-free survival (PFS) and overall survival (OS) were calculated along with the objective response rate (ORR) and disease control rate (DCR).

**Results:**

The study enrolled 217 patients, 58 in the ATC group and 149 in the TC group, and was adjusted to 55 and 118 after PSM, respectively. Both the ORR and DCR were higher in the ATC group compared to the TC group (ORR: 76.4% versus 65.3%; DCR: 89.1% versus 80.1%). After 41.30 months of follow-up, the first-line PFS in the ATC group was significantly longer than in the TC group, while OS was not (PFS: 22.26 months versus 19.18 months, P = 0.02; OS: 42.18 months versus 39.42 months, P = 0.91). Univariate and multivariate analyses indicated that ATC treatment and the absence of brain metastases positively predict PFS, with no variables that dependently predict OS.

**Conclusion:**

The combination of bevacizumab, gefitinib, and chemotherapy significantly benefits patients with the L858R mutation in first-line PFS but not OS.

## 1 Introduction

Lung cancer is one of the most prevalent malignant tumors and the leading cause of tumor-related deaths either worldwide or specifically within the Chinese population (Sung et al., 2021; Han et al., 2024). EGFR-mutated non-small cell lung cancer plays a significant role in the landscape of lung cancer. The L858R mutation in exon 21 and the exon 19 deletion are the most prevalent types of EGFR mutations, constituting around 80%–90% of cases (Hsu et al., 2018). The extensive use of tyrosine kinase inhibitors (TKI) that target mutant EGFR has revolutionized the standards of treatment and management for patients with this disease. The inevitable challenge of resistance to TKI drugs has necessitated the adoption of combination treatment strategies such as chemotherapy to augment the effectiveness of TKIs in patients. A series of clinical trials have confirmed the reliability of chemotherapy in enhancing the efficacy of TKIs (Wu et al., 2013; Hosomi et al., 2020; Planchard et al., 2023). Moreover, the combination of bevacizumab, a VEGFR antibody, with TKIs has also demonstrated efficacy as a treatment approach. Two clinical trials carried out in China and Japan have demonstrated that the initial introduction of Bevacizumab in conjunction with erlotinib extends patients’ progression-free survival (PFS) compared to using erlotinib alone treatment while maintaining acceptable safety profiles (Zhou et al., 2021; Kawashima et al., 2022).

While L858R and 19del variants exhibit sensitivity to TKIs, variations in treatment response exist. Researches indicate that individuals carrying the L858R mutation demonstrate a diminished response to identical treatments (Lee et al., 2013; Sheng et al., 2016). The underlying reason may be owing to the impact of structural variances in the EGFR protein resulting from the mutation site on the affinity for binding to TKI drugs (Kumar et al., 2008). Another pertinent factor to consider is the drug-resistant mutation T790M, which has been observed to exhibit a greater propensity to occur concurrently with L858R (Pao et al., 2005).

The purpose of this study is to investigate whether the addition of Bevacizumab to TKI drugs in combination with chemotherapy, a stronger treatment regimen, can bring more therapeutic benefits for patients with L858R mutation. In the era of precision medicine, providing more evidence-based medicine for EGFR-mutated lung cancer.

## 2 Materials and methods

### 2.1 Study design and patients

Patients who were diagnosed with metastatic non-small cell lung cancer and initiated first-line therapy at the Oncology Department of Shandong Provincial Hospital between June 2019 and December 2021 were retrospectively screened. Clinical characteristics, such as age, gender, smoking status, and clinical stage, are extracted from medical records. The treatment process and efficacy evaluation are retroactively examined. Specifically selected are adult patients with the EGFR exon 21 L858R mutation identified through first- or second-generation sequencing, and an ECOG score of ≥2 in first-line treatment involving the combination of gefitinib and chemotherapy. Patients with concurrent malignant tumors, significant organ dysfunction, mental disorders, substance abuse history, or who died from other causes during follow-up are excluded. Additionally, patients are categorized into two groups based on the use of bevacizumab in the initial treatment regimen: the AGC group receiving anti-angiogenic drugs bevacizumab, gefitinib, and chemotherapy, and the GC group receiving gefitinib and chemotherapy.

### 2.2 Treatment methods

The chemotherapy protocol, incorporating Pemetrexed in conjunction with platinum agents like Carboplatin or Cisplatin, is delivered via intravenous infusion every 3–4 weeks over 4-6 cycles. The administration of pemetrexed and cisplatin was conducted at a dosage of 500 mg and 75 mg per square meter of body surface area, respectively. Carboplatin was administered based on an area under the concentration-time curve of 5 mg/mL. Bevacizumab and chemotherapy are administered concurrently by intravenous injection at a dosage of 15 mg/kg. After completion of the chemotherapy cycle, Bevacizumab is continued for maintenance treatment until disease progression occurs. Gefitinib is administered orally at a dose of 250 mg once daily after confirmation of the L858R mutation in the patient’s genetic testing report.

### 2.3 Definitions and assessments

The response evaluation during treatment is evaluated through imaging studies, including computed tomography (CT) of the neck, chest, and upper abdomen, and magnetic resonance imaging (MRI) scans of the brain. During the chemotherapy cycles, imaging evaluations are conducted before the start of the second cycle of chemotherapy, and then every 2 cycles thereafter. After the completion of chemotherapy, imaging evaluations are conducted every 3 months. For patients without initial brain metastases, brain imaging is performed every 6 months or when clinical symptoms occur. Evaluations were conducted according to the Response Evaluation Criteria in Solid Tumors (RECIST, version 1.1). PFS was measured as the primary endpoint, while the OS, objective response rate (ORR), and disease control rate (DCR) were calculated as the secondary endpoints. Treatment-related adverse events (AEs) were graded and documented according to the Common Terminology Criteria for Adverse Events version 5.0 (CTCAE v5.0), covering hematological and non-hematological toxicities (e.g., general condition, gastrointestinal reactions) as well as targeted therapy-specific reactions (e.g., rash, paronychia, hemorrhage, and venous thrombosis). AE monitoring was conducted throughout each treatment cycle. For patients followed up outside the hospital, standardized telephone interviews combined with electronic medical record systems were used for tracking.

### 2.4 Statistical analysis

The addition of bevacizumab to chemotherapy and gefitinib as a first-line treatment strategy is not a standard clinical practice, marked by a limited sample size within this cohort. Consequently, propensity score matching was employed to mitigate confounding variables. PSM analysis without replacement was conducted using the nearest neighbor method with the setting of a 1:4 matching ratio and a caliper of 0.1, proceeded by the matched package of R (Version 4.3.3). The Clinicopathologic data of patients, including age, ECOG score, smoking status, and metastatic sites were collected and entered into the PSM model. Patients’ baseline data were compared using appropriate statistical methods both before and after matching. Categorical variables were compared using methods like Fisher’s exact test or χ2 test, while continuous variables such as age were assessed using an independent t-test. Time-to-event variables such as the follow-up period and PFS were estimated using the Kaplan-Meier method, and comparisons of variation between curves were assessed using log-rank tests. The Cox proportional-hazards model was carried out to analyze the response and treatment-by-covariate interaction effects of pre-specified patient subgroups as well as for univariate and multivariate analysis to assess the influence of potentially independent prognosis factors on survival functions. Statistical analyses were performed using GraphPad Prism 9. HR with 95% confidence intervals (CI) was presented, and a two-sided *P*-value <0.05 was considered statistically significant.

## 3 Results

### 3.1 Patients’ baseline characteristics

**FIGURE 1:**
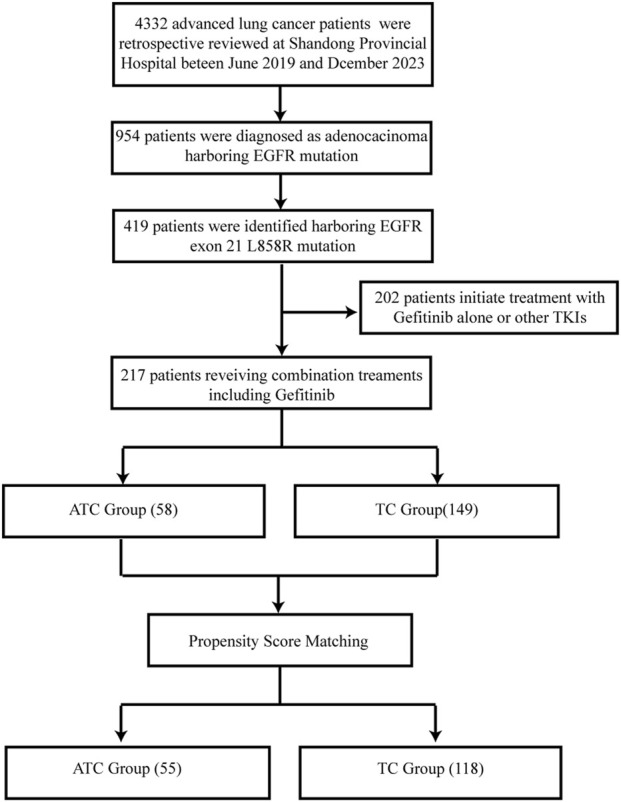
Flow diagram of patients’ enrollment.

Following the commencement of the study, we conducted a retrospective review of 4,332 lung cancer patients’ electronic medical records at Shandong Provincial Hospital between June 2019 and December 2022. Among them, 954 were identified as metastatic lung adenocarcinoma patients with sensitizing EGFR mutations, including 217 with the L858R mutation, who underwent first-line treatment involving a combination therapy with gefitinib. The ATC group consisted of 58 patients, while the TC group included 149 patients. The overall patient enrollment process is shown in Figure 1. Upon propensity score matching, the patient numbers in the two groups were adjusted to 55 and 118, respectively. The baseline clinical characteristics of the patients before and after matching are presented in Table 1. Before matching, the median ages of the ATC group and TC group were 57 and 61 years, respectively, with the majority of patients aged 65 or younger. For the whole population, females and patients with no history of smoking formed the primary composition of both groups. The central nervous system and bones were identified as the predominant sites of metastasis. Before matching, the baseline data displayed statistical differences solely in ECOG scores between the two groups. Processing of PSM balanced clinical characteristics more with no significant variations noted among the diverse variables.

**TABLE 1:** Characteristics of baseline. IQR: interquartile range.

	Before matching			After matching		
	A + T + C	T + C	*P*-value	A + T + C	T + C	*P*-value
	(N = 58)	(N = 149)		(N = 55)	(N = 118)	
Age, median (IQR), years	57.0 [45.0, 78.0]	61.0 [38.0, 76.0]	0.09	57.0 [52.0, 64.0]	59.0 [55.0, 65.0]	0.57
>65 y	13 (22.4%)	34 (22.8%)	0.95	13 (23.6%)	21 (17.8%)	0.37
≤65 y	45 (77.6%)	115 (77.2%)		42 (76.4%)	97 (82.2%)	
Sex			0.88			1.00
Male	20 (34.5%)	48 (32.2%)		19 (34.5%)	41 (34.7%)	
Female	38 (65.5%)	101 (67.8%)		36 (65.5%)	77 (65.3%)	
ECOG score			<0.01			0.06
0	28 (48.3%)	106 (71.1%)		28 (50.9%)	79 (66.9%)	
1	30 (51.7%)	43 (28.9%)		27 (49.1%)	39 (33.1%)	
Smoking Status			0.45			0.57
Never	16 (27.6%)	32 (21.5%)		16 (29.1%)	28 (23.7%)	
Former	42 (72.4%)	117 (78.5%)		39 (70.9%)	90 (76.3%)	
Brain Metastases			0.07			0.34
No	41 (70.7%)	83 (55.7%)		38 (69.1%)	71 (60.2%)	
Yes	17 (29.3%)	66 (44.3%)		17 (30.9%)	47 (39.8%)	
Bone Metastases			0.82			1.00
No	31 (53.4%)	84 (56.4%)		31 (56.4%)	67 (56.8%)	
Yes	27 (46.6%)	65 (43.6%)		24 (43.6%)	51 (43.2%)	
Pleural Effusion or Metastases			0.61			0.77
No	45 (77.6%)	122 (81.9%)		42 (76.4%)	94 (79.7%)	
Yes	13 (22.4%)	27 (18.1%)		13 (23.6%)	24 (20.3%)	
Intrapulmonary Metastasis			0.39			0.67
No	42 (72.4%)	118 (79.2%)		41 (74.5%)	93 (78.8%)	
Yes	16 (27.6%)	31 (20.8%)		14 (25.5%)	25 (21.2%)	
Adrenal Metastasis			0.48			1.00
No	55 (94.8%)	135 (90.6%)		52 (94.5%)	111 (94.1%)	
Yes	3 (5.2%)	14 (9.4%)		3 (5.5%)	7 (5.9%)	
Liver Metastases			0.61			0.73
No	47 (81.0%)	114 (76.5%)		44 (80.0%)	90 (76.3%)	
Yes	11 (19.0%)	35 (23.5%)		11 (20.0%)	28 (23.7%)	
Metastases of Other Sites			0.31			0.80
No	49 (84.5%)	135 (90.6%)		47 (85.5%)	104 (88.1%)	
Yes	9 (15.5%)	14 (9.4%)		8 (14.5%)	14 (11.9%)	

### 3.2 Tumor response

The median follow-up period for the entire population was 41.30 months. In the ATC group, the median follow-up was 41.91 months, while in the TC group, it was 40.89 months. Before PSM, within the first-line treatment, the ATC group witnessed 5 patients achieving complete response (CR), 39 achieving a partial response (PR), and 8 achieving stable disease, whereas in the TC group, these numbers were 7, 94, and 22, respectively. The ORR assessed in the overall cohort amounted to 70.0%, while the DCR reached a notable value of 84.5%. Upon comparing the response rates between the two groups, it was observed that the ATC group exhibited a higher ORR in contrast to the TC group (75.9% versus 67.8%, P = 0.26). The DCR was also higher in the ATC group, with rates of 89.7% compared to 82.6% in the TC group (P = 0.20). Following PSM, the ORR and DCR reached 68.8% and 83.2%, respectively. While the ATC group exhibited higher ORR (76.4% versus 65.3%, P = 0.14) and DCR (89.1% versus 80.1%, P = 0.16) values compared to the TC group, the difference was not statistically significant among the matched pairs (Table 2).

**TABLE 2:** Summary of survival analysis and therapeutic effects of the two groups.

	Before matching			After matching		
	ATC group	TC group	*P*-value	ATC group	TC group	*P*-value
	(N = 58)	(N = 149)		(N = 55)	(N = 118)	
mPFS (month)	22.18	16.16	<0.01	22.26	19.18	0.02
PFS Rate (%)						
6 months	96.6	85.9	0.06	98.2	84.7	0.02
12 months	81.8	61.7	0.05	78.2	64.4	0.09
18 months	61.8	43.0	0.06	58.2	50.0	0.38
mOS (month)	42.18	39.42	0.75	43.99	43.54	0.91
OS Rate (%)						
12 months	98.3	93.3	0.12	98.2	93.2	0.13
24 months	69.0	68.5	0.94	69.1	73.7	0.45
36 months	46.6	45.0	<0.01	43.6	49.2	0.43
ORR (%)	75.9	67.8	0.26	76.4	65.3	0.14
DCR (%)	89.7	82.6	0.20	89.1	80.1	0.16
Best response (case)						
Complete response	5	7	0.45	4	6	0.82
Partial response	39	94	0.58	38	71	0.26
Stable disease	8	22	0.86	7	18	0.66

### 3.3 Progression-free survival

Before PSM, a total of 164 progress events (79.2%) were observed, with 42 patients (72.4%) in the ATC group and 122 patients (81.9%) in the TC group. The median PFS of the entire cohort was 18.77 months, whereas it was 22.18 months in the ATC group, showing a statistical difference compared to 16.16 months in the TC group (P < 0.01) (Supplementary Figure S1A). The PFS rates of the patients in the ATC group at the 6th, 12th, and 18th months were higher than that in the TC group, but no statistical differences were observed (Table 2).

**FIGURE 2:**
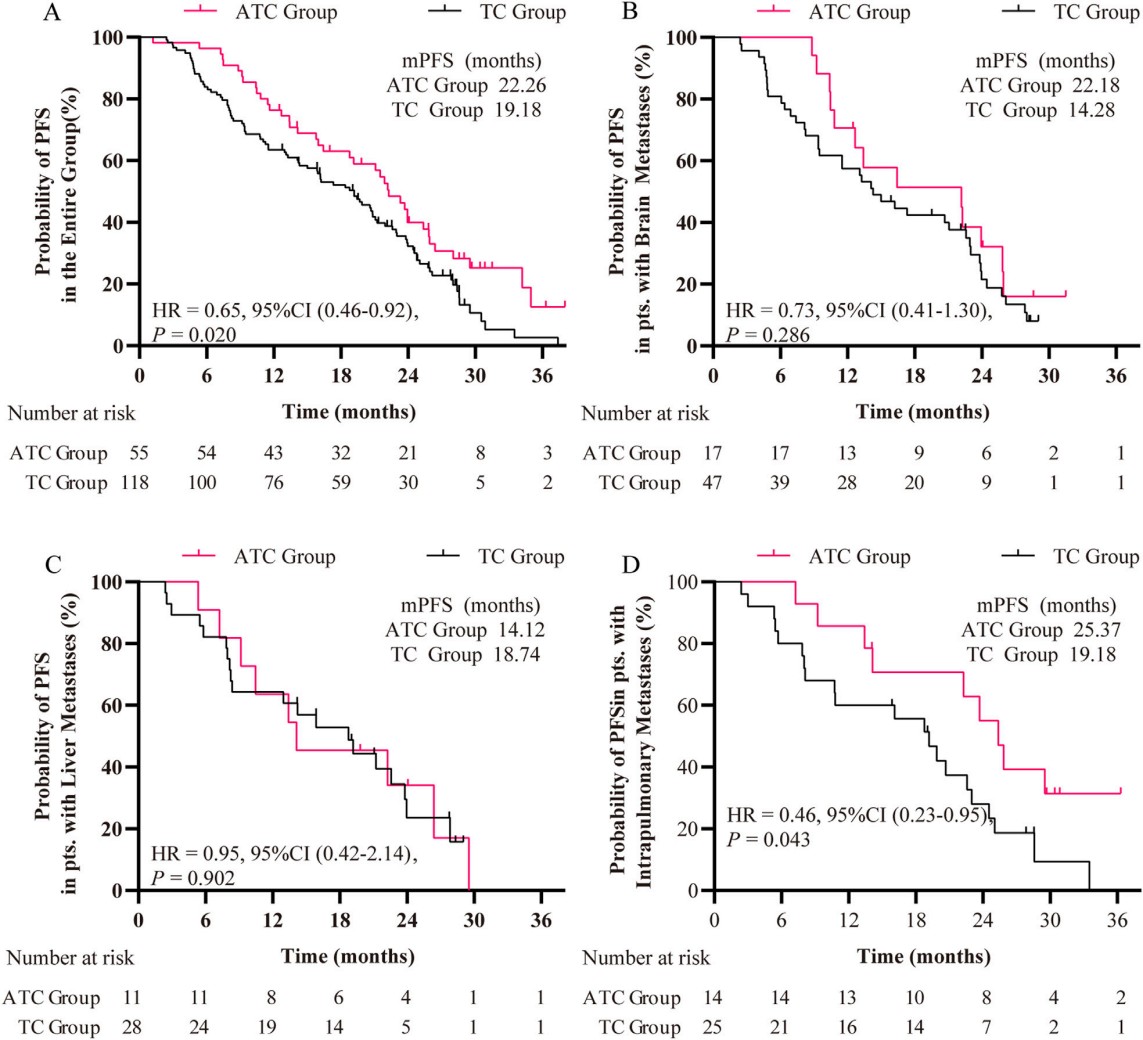
Kaplan-Meier curves of first-line PFS of patients **(A)** in the entire group, **(B)** in patients with brain metastases, **(C)** in patients with liver metastases, and **(D)** in patients with intrapulmonary metastases. HR: hazard ratio.

After PSM, the first-line PFS of patients in the ATC group demonstrated a significant improvement over that of the TC group patients (22.26 months versus 19.18 months, P = 0.02) (Figure 2A). Additionally, the PFS rates of the ATC group at the 6th, 12th, and 18th months (98.2%, 78.2%, 58.2%) exceeded those of the TC group (P < 0.05 for the 6th month) (Table 2). We also evaluated the differences in treatment outcomes in subgroups of patients with brain metastases, liver metastases, and intrapulmonary metastases. In patients with intrapulmonary metastases, the PFS was 25.37 months in the ATC group and 19.18 months in the TC group, showing a statistically significant difference (P = 0.036) (Figure 2B). However, no significant differences were observed in the subgroups of patients with central nervous system metastases (P = 0.286) and liver metastases (P = 0.902) (Figures 2C,D). The Cox regression model was used to analyze various statistical factors through univariate and multivariate analysis. The results showed that the ATC treatment regimen and brain metastasis were independent protective factors for first-line PFS in patients with the L858R mutation, providing evidence for the analysis of PFS results using the Kaplan-Meier method (Table 3).

**TABLE 3:** Univariate and multivariable analysis for first-line PFS.

	Univariate analysis		Multivariate analysis	
	HR (95%CI)	*P*-value	HR (95%CI)	*P*-value
Age				
≤65 y	1	0.81	1	0.87
>65 y	1.06 (0.68–1.63)		1.04 (0.65–1.66)	
Sex				
Male	1	0.96	1	0.99
Female	0.99 (0.69–1.42)		1.00 (0.60–1.69)	
Smoking status				
Never	1	0.97	1	0.67
Former	1.01 (0.69–1.48)		0.88 (0.51–1.55)	
ECOG-PS				
0	1	0.82	1	0.40
1	0.96 (0.67–1.37)		1.17 (0.81–1.68)	
Treatment				
ATC Group	1	0.02	1	0.02
TC Group	1.59 (1.09–2.33)		1.60 (1.09–2.35)	
Brain metastases				
Yes	1	0.03	1	0.03
No	0.68 (0.48–0.97)		1.51 (1.03–2.21)	
Pleural metastases or effusion				
Yes	1	0.86		
No	0.97 (0.65–1.44)			
Bone metastasis				
Yes	1	0.24		
No	1.23 (0.87–1.73)			
Itrapulmonary metastasis				
Yes	1	0.54		
No	1.14 (0.76–1.71)			
Adrenal metastasis				
Yes	1	0.08	1	0.08
No	0.55 (0.28–1.09)		1.94 (0.93–4.08)	
Liver metastasis				
Yes	1	0.34		
No	0.82 (0.54–1.24)			
Metastasis of other sites				
Yes	1	0.86		
No	0.96 (0.57–1.59)			

### 3.4 Overall survival

**FIGURE 3:**
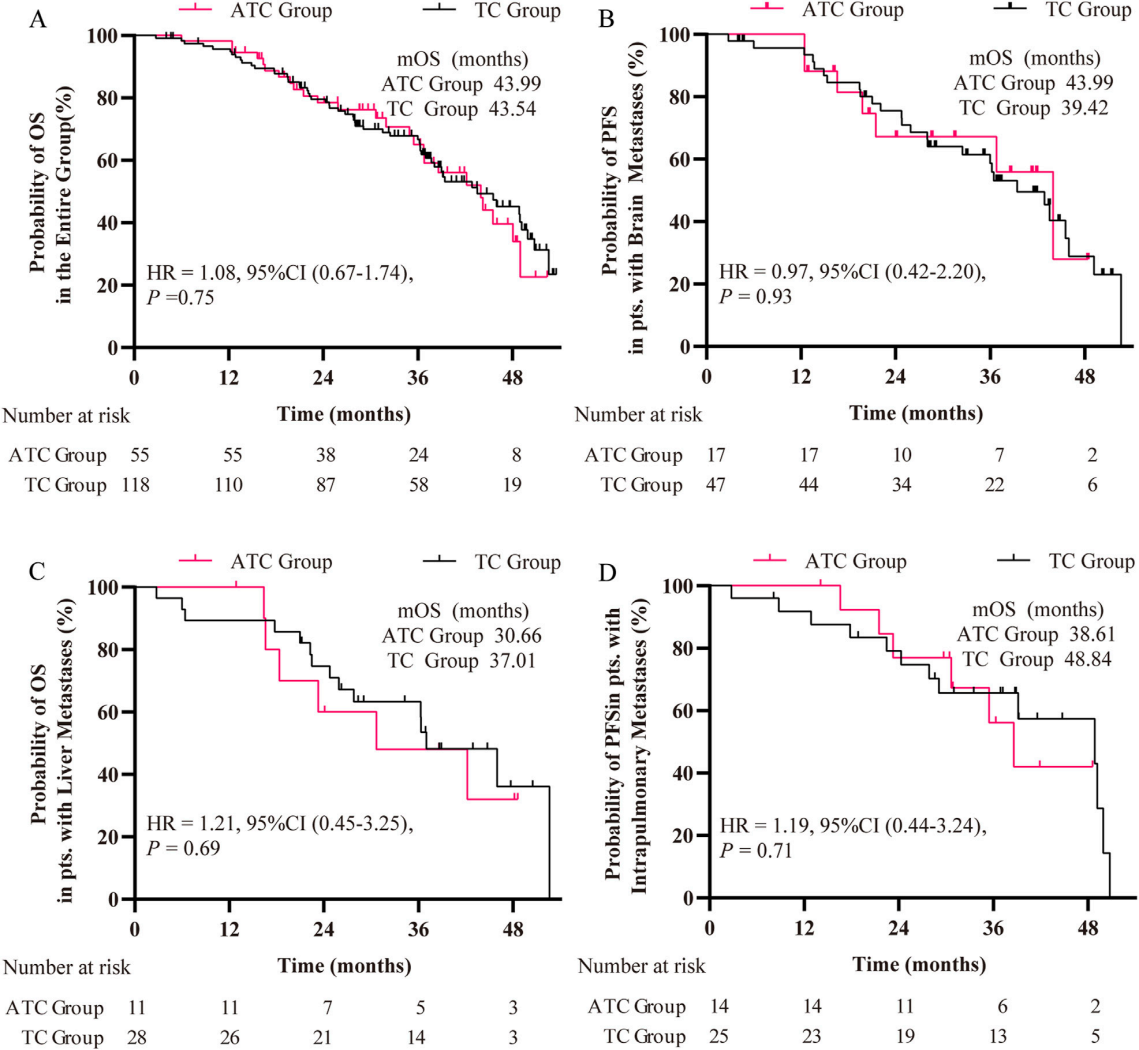
Kaplan-Meier curves of OS of patients **(A)** in the entire group, **(B)** in patients with brain metastases, **(C)** in patients with liver metastases, and **(D)** in patients with intrapulmonary metastases. HR: hazard ratio.

At the date of cut-off, a total of 106 death events (79.2%) were observed for the whole population, with 28 patients (72.4%) in the ATC group and 78 patients (81.9%) in the TC group. The median OS of the entire cohort was 39.42 months, but no significant difference was observed between the ATC and TC groups (42.18 months versus 39.42 months, HR = 0.98, P = 0.91) (Supplementary Figure S1B). Following PSM, while the median OS of patients in the ATC group continues to exceed that of the TC group, no statistically significant difference exists between the survival curves (43.99 months versus 43.54 months, HR = 1.08, P = 0.75) (Figure 3A).

Regarding OS rate, prior to PSM, the ATC group exhibited higher OS rates than the TC group at the 12th, 24th, and 36th months, with a significant difference observed at 36th months (P < 0.01) (Table 2). Following PSM, the OS rate of the ATC group surpassed that of the TC group at 12 months, but was lower at 24 and 36 months, with no statistically significant difference (Table 2).

When evaluating the impact of the two treatment regimens on OS in subgroups of patients with brain metastases, liver metastases, and lung metastases after PSM, no statistical differences were observed (Figures 3B–D). After Cox univariate and multivariable regression analyses, no independent factors influencing the OS of patients with the L858R mutation were found after univariate and multivariate analysis (Table 4).

**TABLE 4:** Univariate and multivariable analysis for OS.

	Univariate analysis		Multivariate analysis	
	HR (95%CI)	*P*-value	HR (95%CI)	*P*-value
Age				
≤65 y	1	0.75	1	0.73
>65 y	1.1 (0.63–1.92)		1.106 (0.629–1.944)	
Sex				
Male	1	0.42	1	0.88
Female	1.21 (0.76–1.92)		0.951 (0.49–1.843)	
Smoking status				
Never	1	0.48	1	0.47
Former	1.21 (0.71–2.04)		1.293 (0.647–2.582)	
ECOG-PS				
0	1	0.56		
1	1.14 (0.73–1.79)			
Treatment				
ATC Group	1	0.75	1	0.15
TC Group	0.92 (0.57–1.49)		0.707 (0.442–1.131)	
Brain metastases				
Yes	1	0.08	1	0.04
No	1.48 (0.95–2.31)		0.61 (0.383–0.97)	
Pleural metastases or effusion				
Yes	1	0.37		
No	0.78 (0.45–1.35)			
Bone metastases				
Yes	1	0.37		
No	0.82 (0.52–1.27)			
Intrapulmonary metastasis				
Yes	1	0.55		
No	1.17 (0.7–1.96)			
Adrenal metastasis				
Yes	1	0.03		
No	2.24 (1.07–4.7)			
Liver metastasis				
Yes	1	0.2		
No	1.38 (0.84–2.28)			
Metastasis of other sites				
Yes	1	0.97		
No	0.99 (0.51–1.92)			

### 3.5 Saftey

Table 5 summarizes the incidence of AEs during treatment in both groups after PSM. Various grades of myelosuppression were the most common AEs in this study. In the ATC group, the incidence of grade ≥3 anemia, leukopenia, neutropenia, and thrombocytopenia was 12.7%, 12.7%, 10.9%, and 9.1%, respectively, which was comparable to the TC group. However, the incidence of grade 1–2 myelosuppression events was higher in the ATC group. Regarding effects on liver and kidney function, the overall incidence of AEs was similar between the two groups, but more severe liver dysfunction was more common in the ATC group (10.9% vs. 1.7%). The incidence of treatment-related gastrointestinal disorders, such as decreased appetite, nausea, vomiting, and diarrhea, was generally similar between the two groups. Oral mucositis, rash, and paronychia are characteristic AEs associated with gefitinib. In this study, the combination of bevacizumab and gefitinib did not increase the incidence of oral mucositis or paronychia but did increase the incidence of rash (grade 1–2: 69.1%; grade ≥3: 10.9%). Due to the addition of bevacizumab, three patients (5.3%) in the ATC group developed grade ≥3 hypertension, and one patient (1.8%) experienced hemoptysis. No AE-related deaths occurred in this study. Overall, the combination of bevacizumab, gefitinib, and chemotherapy led to an increased incidence of AEs across all grades, but these remained within an acceptable range.

**TABLE 5:** Treatment-emergent adverse events.

	A + T + C (N = 55)		T + C (N = 118)	
	All grades	Grade ≥3	All grades	Grade ≥3
Anemia	29 (52.7%)	7 (12.7%)	56 (47.5%)	13 (11.0%)
Leukopenia	31 (56.4%)	7 (12.7%)	61 (51.7%)	15 (12.7%)
Neutropenia	27 (49.1%)	6 (10.9%)	51 (43.2%)	12 (10.2%)
Thrombocytopenia	23 (41.8%)	5 (9.1%)	43 (36.4%)	7 (5.9%)
Hepatic aminotransferase increased	30 (54.5%)	6 (10.9%)	58 (49.2%)	2 (1.7%)
Renal Dysfunction	17 (30.9%)	3 (5.3%)	36 (30.5%)	4 (3.4%)
Decreased appetite	25 (45.5%)	2 (3.7%)	42 (35.6%)	3 (2.5%)
Vomiting	15 (27.3%)	2 (3.7%)	28 (23.7%)	1 (0.8%)
Nausea	21 (38.2%)	4 (7.3%)	48 (40.7%)	7 (5.9%)
Diarrhea	21 (38.2%)	3 (5.3%)	50 (42.4%)	5 (4.2%)
Stomatitis	20 (36.4%)	1 (1.8%)	36 (30.5%)	2 (1.7%)
Rash	36 (65.5%)	6 (10.9%)	68 (57.6%)	6 (5.1%)
Paronychia	16 (29.1%)	1 (1.8%)	28 (23.7%)	1 (0.8%)
Asthenia	33 (60.0%)	6 (10.9%)	53 (44.9%)	9 (7.6%)
Hypertension	27 (49.1%)	3 (5.3%)	28 (23.7%)	-
Hemorrhage	15 (27.3%)	1 (1.8%)	18 (15.3%)	-
Superficial vein thrombosis	9 (16.4%)	-	10 (8.5%)	-

## 4 Discussion

This retrospective study demonstrates that first-line administration of the anti-angiogenic agent bevacizumab combined with chemotherapy and TKIs in advanced NSCLC patients harboring EGFR-L858R mutations improves ORR and DCR, resulting in significantly prolonged PFS. To our knowledge, this represents the first investigation evaluating the efficacy of a comprehensive regimen integrating anti-angiogenic therapy, chemotherapy, and TKIs as initial treatment for this patient population.

L858R and 19Del constitute the predominant EGFR mutation subtypes sensitive to TKIs (Hsu et al., 2018). While both mutations exhibit differential clinical responses to TKI therapy, it is well established that patients harboring the L858R mutation experience inferior clinical outcomes (Lee et al., 2015; Sheng et al., 2016; Chan et al., 2022). Mechanistic insights into this differential response include structural and functional divergences between the two mutants. The variant protein structures exhibit distinct conformational changes, resulting in differential TKI-binding affinity. Drug-binding kinetics analyses confirm higher binding affinity of TKIs to the 19del mutant versus L858R (Carey et al., 2006). Additionally, EGFR amplification levels and phosphorylation sites exhibit inter-subtype heterogeneity, leading to differential downstream pathway activation (Okabe et al., 2007). Notably, L858R demonstrates significantly higher co-occurrence rates with compound EGFR mutations compared to 19del, potentially contributing to primary TKI resistance mechanisms (Zhao et al., 2023).

The development of tyrosine kinase inhibitors (TKIs) has transformed the management of non-small cell lung cancer (NSCLC) with epidermal growth factor receptor (EGFR) mutations. TKIs also show promise as nuclear medicine imaging tracers for NSCLC diagnosis (Fawwaz et al., 2024). Nevertheless, curative outcomes remain elusive due to population heterogeneity along with inevitable drug resistance challenges. In cases where side effects can be tolerated, combination therapy is preferred and is more effective in slowing down the progression of the disease. The commonly used TKI drugs have been confirmed to provide significantly higher benefits when used in combination with chemotherapy compared to single-agent therapy (Sequist et al., 2013; Wu et al., 2013; Hosomi et al., 2020; Planchard et al., 2023). Three randomized trials in Asian advanced NSCLC populations demonstrated that first-line platinum-based chemotherapy concurrent with gefitinib achieved PFS ranging from 15.8 to 20.9 months. Notably, the NEJ005 and NEJ009 trials reported median OS of 41.9 and 50.9 months, respectively (Sugawara et al., 2015; Cheng et al., 2016; Hosomi et al., 2020). In our cohort, patients receiving gefitinib plus chemotherapy exhibited a median PFS of 19.18 months and a median OS of 43.54 months, aligning with published findings.

Preclinical studies demonstrate significant functional crosstalk and synergistic effects between the EGFR and vascular endothelial growth factor (VEGF) pathways in NSCLC, evidenced both *in vitro* and *in vivo* (Le et al., 2021). EGFR and VEGF signaling converge on downstream pathways including RAS/RAF/MAPK and PI3K/AKT/mTOR (Osude et al., 2022). Transcriptional profiling reveals coordinated upregulation of VEGF and VEGFR-2 in EGFR-mutant tumors, suggesting bidirectional pathway crosstalk at transcriptional and translational levels (Hung et al., 2016). Pharmacologically, VEGFR-targeted agents enhance intratumoral penetration of small-molecule TKIs. Gefitinib notably suppresses VEGFR expression through both HIF-1–dependent and HIF-1–independent mechanisms, amplifying antitumor efficacy (Wildiers et al., 2003; Pore et al., 2006). In EGFR-mutant NSCLC cell lines, TKIs like erlotinib suppress VEGF transcription by destabilizing HIF-1 (Ciardiello et al., 2001). And erlotinib combined with bevacizumab reduces tumor microvessel density, inhibits tumor growth, and enhances antitumor efficacy versus monotherapy in EGFR-mutant xenografts (Naumov et al., 2009; Masuda et al., 2017).

Moreover, integrating bevacizumab with TKI therapy yields additional clinical benefits through synergistic suppression of tumor proliferation and metastasis. A Japanese trial demonstrated superior PFS with bevacizumab plus erlotinib versus erlotinib monotherapy (16.9 vs. 13.3 months; P = 0.016), without a significant OS difference (50.7 vs. 46.2 months; P = 0.97) (Saito et al., 2019; Kawashima et al., 2022). Similarly, a Chinese trial confirmed that simultaneous VEGFR and EGFR inhibition improves first-line PFS (17.9 vs. 11.2 months; P < 0.001), with particular benefit in L858R-mutant patients (19.5 vs. 9.7 months; P = 0.001) (Zhou et al., 2021). Our data revealed that the bevacizumab/gefitinib/chemotherapy regimen significantly improved first-line PFS compared to gefitinib/chemotherapy alone (22.26 vs. 19.18 months; P = 0.02), though without significant OS extension (43.35 vs. 43.54 months; P = 0.91).

Most lung cancer patients present with distant metastases, a primary contributor to the disease’s high mortality rate. Brain metastases, occurring in approximately 30% of EGFR-mutant patients, are independently associated with poorer prognosis (Baik et al., 2015; Dormieux et al., 2020). Our analysis further identifies brain metastasis as an independent negative prognostic factor in L858R-mutant patients. Gefitinib, a first-generation EGFR-TKI, demonstrates limited blood-brain barrier (BBB) penetration, restricting its efficacy against intracranial lesions (Ballard et al., 2016; Wrona et al., 2018). A multicenter phase 3 trial revealed that chemotherapy combined with gefitinib significantly prolongs intracranial PFS, systemic PFS, and OS in patients with brain metastases (Hou et al., 2023). In this study, median PFS was 22.18 months in the ATC group versus 14.17 months in the TC arm (p = NS), suggesting equivalent therapeutic robustness. The absence of additional benefit from bevacizumab indicates non-synergistic activity with this TKI-chemotherapy backbone. Notably, our data demonstrate that incorporating bevacizumab in patients with pulmonary metastases significantly extended PFS when added to gefitinib plus chemotherapy (25.37 vs. 19.18 months; P = 0.043), indicating enhanced responsiveness relative to other metastatic sites - consistent with prior reports (Lin et al., 2016).

Regarding treatment safety assessment, although the ATC regimen increased the risk of specific AEs due to the inclusion of bevacizumab, the majority of treatment-related toxicities remained controllable through dynamic monitoring and graded management strategies. Previous Phase III clinical data in East Asian NSCLC populations reported that gefitinib combined with platinum-based doublet chemotherapy induced myelosuppression (including leukopenia, anemia, and thrombocytopenia) with overall incidence rates ranging from 20% to 60%, and Grade 3–4 hematological toxicities occurring in approximately 10%–30% (Yoshimura et al., 2015; Han et al., 2017; Hosomi et al., 2020). In this study, the observed incidence of hematological toxicities—anemia (52.7%), leukopenia (56.4%), neutropenia (49.1%), and thrombocytopenia (41.8%)—was generally consistent with or slightly lower than those reported in the aforementioned prospective studies. Notably, skin toxicity, a characteristic AE of TKIs, showed a high cumulative incidence in this cohort (ATC group 65.5% vs TC group 57.6%). The increased incidence may be related to bevacizumab-induced vascular endothelial dysfunction and IgE-mediated delayed hypersensitivity. Additionally, bevacizumab-specific vascular toxicities were observed in the ATC group, including Grade 3 hypertension (3 cases, 5.3%) and Grade 3 bleeding (1 case, 1.8%), while no such severe AEs were recorded in the TC group. Compared with safety data from registered clinical trials such as NEJ026, JO25567, and ARTEMIS-CTONG1509, the incidence of Grade 3 hypertension (5.3%) and bleeding events (1.8%) in this study was relatively low, and overall toxicity remained within an acceptable range (Seto et al., 2014; Hosomi et al., 2020; Zhou et al., 2021). It should be noted that, due to the retrospective nature of real-world data collection, the prophylactic use of supportive medications (e.g., G-CSF, recombinant human thrombopoietin) may have mitigated hematological toxicity severity. Meanwhile, recall bias regarding non-severe AEs might have led to underreporting of low-grade toxicities. These factors collectively represent potential reasons for the lower incidence of AEs observed in this study compared to previous clinical trials.

This study has several limitations. First, its single-center retrospective design precludes external validation and introduces inherent confounding biases despite covariate adjustment via propensity score matching (PSM). Second, the sample size remains limited due to infrequent clinical adoption of the bevacizumab/chemotherapy/gefitinib combination per current guidelines. Third, though economic factors critically influence treatment decisions, fluctuating drug pricing and dynamic reimbursement policy adjustments by China’s National Healthcare Security Administration precluded formal cost-effectiveness analysis.

In conclusion, this study demonstrates that adding bevacizumab to gefitinib and chemotherapy was associated with improved PFS in patients with advanced EGFR L858R-mutant NSCLC, though without conferring a corresponding OS benefit. The exploratory nature of these results warrants prospective validation before clinical implementation. As novel agents enter the therapeutic landscape, rigorous investigation of optimized combination strategies will advance precision oncology paradigms. Scientifically robust validation through prospectively designed, large-scale randomized controlled trials remains imperative for substantiating these combinatorial approaches.

## Data Availability

The raw data supporting the conclusions of this article will be made available by the authors, without undue reservation.

## Abbreviations

NSCLC, Non-small cell lung cancer; EGFR, Epidermal Growth Factor Receptor; TKI, Tyrosine kinase inhibitor; PSM, propensity score matching; PFS, Progression-free survival; OS: Overall survival; ORR, Objective response rate; DCR: Disease control rate; CR, Complete response; PR, Partial response; SD, Stable disease. HR, Hazard ratio IQR, Interquartile range; CI, Confidence interval.
