# Supplementary figures and images for: Addition of bevacizumab to gefitinib plus chemotherapy as first-line therapy in EGFR L858R mutate advanced non-small cell lung cancer patients
Source: Front Pharmacol. 2025 Oct 8;16:1503171. doi: 10.3389/fphar.2025.1503171 (PMC12541177; doi:10.3389/fphar.2025.1503171)

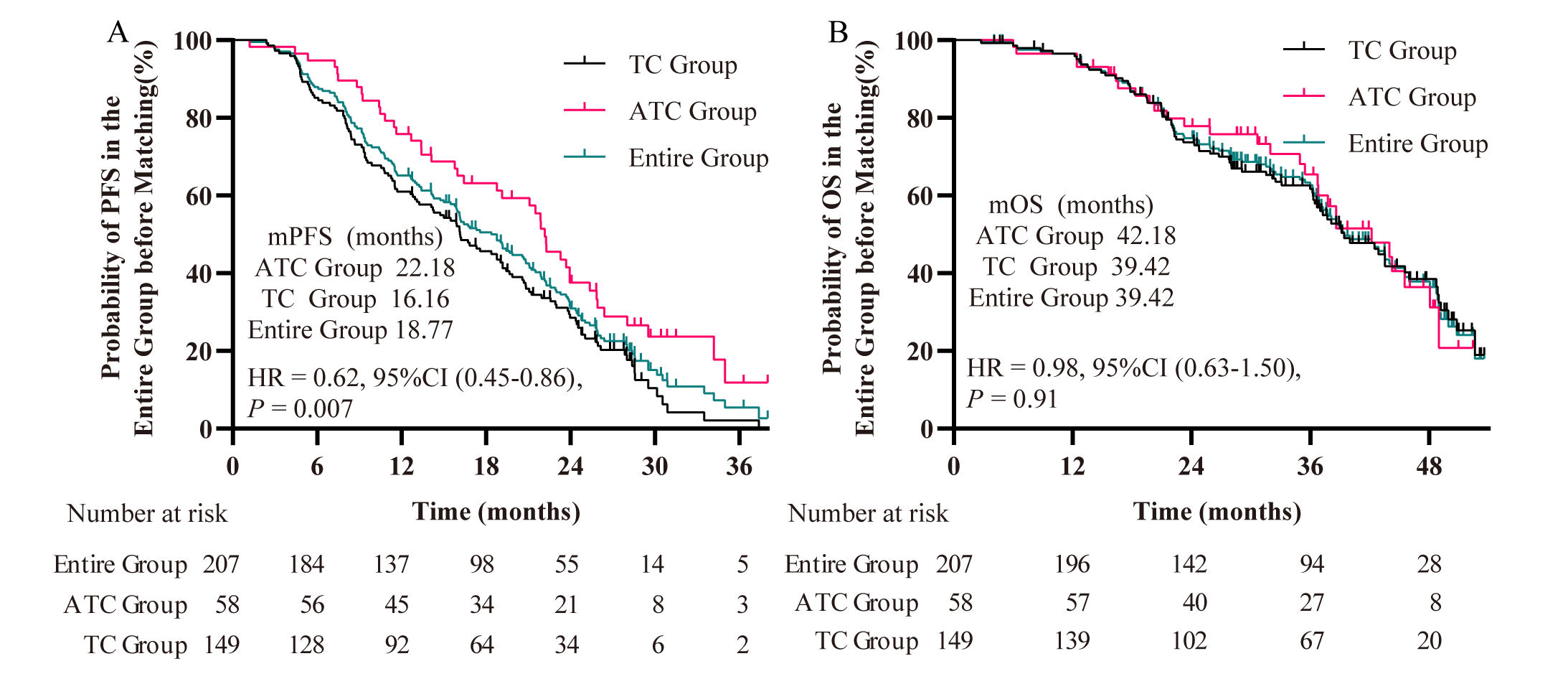

Supplement: Supplementary file 1 [file Image1.tif]
